# Inhaled Tranexamic Acid for Massive Hemoptysis in the Setting of Oral Anticoagulation: A Case Report

**DOI:** 10.5811/cpcem.2020.7.48525

**Published:** 2020-10-05

**Authors:** Jacqueline M. Dempsey, Mandy Jones, Jonathan Bronner, Connor Greer, Gavin T. Howington

**Affiliations:** * University of Kentucky HealthCare, Department of Pharmacy Services, Lexington, Kentucky; † University of Kentucky, Department of Pharmacy Practice and Science, Lexington, Kentucky; ‡ University of Kentucky HealthCare, Department of Emergency Medicine, Lexington, Kentucky

**Keywords:** rivaroxaban, hemoptysis, tranexamic acid

## Abstract

**Introduction:**

We discuss a case of massive hemoptysis in the setting of a direct-acting oral anticoagulant (DOAC) successfully managed with nebulized tranexamic acid (TXA).

**Case Report:**

Per the American College of Cardiology and the American Society of Hematology, it is recommended that significant bleeding associated with a DOAC be treated with either 4-factor prothrombin complex concentrate or andexanet alfa. However, our patient was at high risk for thrombotic complications given a recent pulmonary embolism.

**Conclusion:**

We demonstrate that it is reasonable to trial nebulized TXA given its low cost, ease of administration, and safety profile. Additionally, this report discusses a unique dosing strategy and a previously unreported complication associated with nebulization of undiluted TXA.

## INTRODUCTION

Hemoptysis is the expectoration of blood that originates from the airway, and can be classified as either non-massive or massive in nature.^1^ Massive hemoptysis lacks a clear definition but is often considered to be greater than 200 milliliters (mL) per day.^1^,^2^ Mortality rates vary based on etiology and comorbidities; however, hemoptysis is typically considered a medical emergency secondary to the risk for asphyxiation.^1^ Tranexamic acid (TXA) is an antifibrinolytic agent that prevents degradation of existing fibrin clots. It is a lysine analog that forms a reversible complex with plasminogen, displacing it from fibrin, resulting in the inhibition of fibrinolysis. Additionally, TXA inhibits the proteolytic activity of plasmin.^3^ Intravenous and oral TXA have been studied in the setting of hemoptysis with inconsistent results; however, a promising trial was recently published on the use of nebulized TXA.^2^,^4^,^5^

Few attempts have been made to inhibit bleeding related to direct oral anticoagulants (DOAC) with TXA, and those cases published demonstrate variable results.^6^–^8^ Many published cases of hemoptysis in the setting of rivaroxaban use were resolved by holding anticoagulation; however, these reports were not designated as massive hemoptysis and did not mention consideration of anticoagulation reversal.^9^,^10^ There is limited evidence discussing the use of nebulized TXA for management of massive hemoptysis in the setting of anticoagulation with a DOAC.

The relatively benign side-effect profile of nebulized TXA makes it a low-risk pharmacologic option for the management of hemoptysis. The use of this agent has been briefly investigated for both hemoptysis and post-tonsillectomy bleeding. To our knowledge, the use of TXA for management of hemoptysis in the setting of a DOAC has been reported only minimally. We describe the use of nebulized TXA in a medically complex patient receiving a DOAC to avoid complications associated with hemoptysis as well as the need for administration of 4-factor prothrombin complex concentrate (4F-PCC).

## CASE REPORT

A 65-year-old male presented to the emergency department (ED) via emergency medical services (EMS) for evaluation secondary to a possible gastrointestinal bleed. Per EMS, approximately 300 mL of frank blood were noted in a bucket located in the patient’s home. Soon after arriving to the ED the patient was found to be experiencing massive hemoptysis. The patient’s vital signs on arrival included a blood pressure of 135/80 millimeters of mercury, a heart rate of 104 beats per minute, and respiratory rate of 35 breaths per minute. Additionally, his oxygen saturation was 92% on room air. Pertinent past medical history included chronic obstructive pulmonary disease, left upper lobe pulmonary mass concerning for malignancy or infection, heart failure, and tobacco use. Additionally, the patient had been discharged from the hospital four days prior with a new prescription for rivaroxaban 15 milligrams (mg) by mouth twice daily for 21 days, followed by 20 mg once daily after being diagnosed with a subsegmental pulmonary embolism (PE). Enoxaparin had been transitioned to rivaroxaban while inpatient, and outpatient adherence to rivaroxaban was confirmed.

The patient continued to expectorate a large volume of blood, and there was concern for impending airway compromise. Prior to consideration of 4F-PCC, the decision was made to trial nebulized TXA. An intravenous solution of TXA 500 mg was initially nebulized directly with a Hudson RCI Micro Mist nebulizer (Teleflex Incorporated, Wayne, PA) at an oxygen flow rate of 8 L per minute. When using undiluted TXA, the solution began to crystallize, preventing further nebulization, which has not previously been reported. Subsequently, respiratory therapy added 3 mL of 0.9% sodium chloride, resulting in a final concentration of 62.5 mg/mL). Nebulization was completed successfully in approximately 10 minutes. Shortly after administration, the volume and frequency of hemoptysis significantly decreased with only occasional blood-tinged sputum. No systemic reversal of anticoagulation was required. Notable labs on admission included an elevated international normalized ratio of 2.9 (reference range 0.9–1.1) likely secondary to malnutrition and possibly rivaroxaban use, as well as an anti-factor Xa level > 2.0 international units/mL.^11^

The patient was subsequently admitted to the pulmonary intensive care unit and continued TXA 500 mg nebulized every eight hours for a total of three doses. A diagnostic bronchoscopy was never performed per patient request. Anticoagulation was held throughout the admission, and bleeding ultimately subsided. The patient was discharged two days after admission and anticoagulation was not resumed.

CPC-EM Capsule

What do we already know about this clinical entity?

*Nebulized tranexamic acid (TXA) has demonstrated promising results when used to treat hemoptysis*.

What makes this presentation of disease reportable?

*A patient presenting with massive hemoptysis exacerbated by direct-acting oral anticoagulant (DOAC) was treated with nebulized TXA*.

What is the major learning point?

*Consideration of nebulized TXA for the treatment of massive hemoptysis is reasonable for patients on DOACs*.

How might this improve emergency medicine practice?

*When treating hemoptysis exacerbated by anticoagulation with a DOAC, nebulized TXA may lead to rapid resolution and less need for anticoagulation reversal*.

## DISCUSSION

We have reported a case of massive hemoptysis in the setting of rivaroxaban therapy managed with nebulized tranexamic acid. A recently published randomized, controlled trial evaluated the use of TXA 500 mg nebulized every eight hours for up to five days in 55 patients.^2^ When compared to placebo, nebulized TXA was superior in decreasing the amount of expectorated blood starting at day two of treatment as well as the duration of bleeding at day five. Approximately 57% of patients included in this study were on an anticoagulant or an antiplatelet agent. However, only one of these patients was receiving a DOAC. Additionally, patients with massive hemoptysis, defined as greater than 200 mL, were excluded making the trial less applicable to our patient.

Per the American College of Cardiology and the American Society of Hematology, it is recommended that significant bleeding associated with a DOAC be treated with either 4F-PCC or andexanet alfa.^12^,^13^ Andexanet alfa is not a formulary agent at our institution. Thus, we use 4F-PCC when pharmacologic intervention is deemed necessary for management of bleeding in the setting of a DOAC. Given the patient’s possible malignancy and recent PE, there was concern regarding his prothrombotic risk.^14^ Additionally, 4F-PCC is significantly more expensive than TXA. Therefore, we opted to trial nebulized TXA due to its safety profile and low cost.

Per our literature search, one case of hemoptysis in the setting of rivaroxaban use was successfully managed with nebulized TXA.^8^ This case described a patient with a similar presentation of massive hemoptysis, which was attributed to stage IV adenocarcinoma. The patient had recently been diagnosed with a PE and was receiving active treatment with rivaroxaban. The authors noted concern for diffuse alveolar hemorrhage and proceeded with nebulized TXA 1000 mg (50 mg/mL) to avoid the need for systemic reversal of anticoagulation. Immediate symptom resolution was reported, and the patient did not receive any subsequent doses of TXA. An additional case report of cancer-related, non-massive hemoptysis described using a dose of TXA 1000 mg nebulized over 45 minutes.^15^ We describe a case of massive hemoptysis in the setting of anticoagulation with a DOAC successfully treated with nebulized TXA at a dose of 500 mg.

Nebulized TXA can be prepared and administered quickly, allowing for rapid reassessment of the need for further interventions. The initial dose of TXA was completed within approximately 10 minutes of initiation, and significant improvement of symptoms was noted immediately after completion. Several concentrations of TXA have been documented in the literature as successfully nebulized, ranging from 10mg/mL to 100mg/mL.^2^,^8^,^15^ The combination of undiluted tranexamic acid 100 mg/mL and the Micro Mist nebulizer resulted in crystallization that slowed the nebulization process. However, crystallization resolved after the addition of 3 mL of 0.9% sodium chloride. Currently, we do not have a clear explanation for this issue.

## CONCLUSION

We report a case of massive hemoptysis in the setting of rivaroxaban use successfully treated with nebulized TXA. These results are consistent with the growing body of literature surrounding this topic. In the case of massive hemoptysis where rapid resolution is essential, it is reasonable to consider nebulized TXA prior to initiation of systemic anticoagulation reversal when indicated. Further investigation is needed to determine optimal dosing and concentration of TXA. At this time, emergency providers should be aware of the possibility of crystallization if using undiluted TXA.
